# Comparison of intestinal ischemia after on-pump versus off-pump coronary artery bypass grafting surgery

**DOI:** 10.3906/sag-1705-131

**Published:** 2019-02-11

**Authors:** Lütfi SOYLU, Oğuz Uğur AYDIN, Mehmet YILDIZ, Hacer SERDAROĞLU, Murat KURTOĞLU, Sedat KARADEMİR

**Affiliations:** 1 Department of General Surgery, Ankara Güven Hospital, Ankara Turkey; 2 Department of Anesthesiology and Reanimation, Ankara Güven Hospital, Ankara Turkey; 3 Department of Cardiovascular Surgery, Ankara Güven Hospital, Ankara Turkey

**Keywords:** Mesenteric ischemia, cardiac surgery, risk factors, complication

## Abstract

**Background/aim:**

Acute mesenteric ischemia (AMI), one of the gastrointestinal system complications, which occurs following cardiac surgery, is challenged in the literature with a diminished incidence of AMI by heart surgery without cardiopulmonary bypass (CPB) or with pulsatile CPB. This study aims to compare the incidence and mortality rate of mesenteric ischemia in a series of consecutive patients undergoing coronary artery bypass grafting (CABG) through on-pump and off-pump techniques.

**Materials and methods:**

This study included patients who underwent CABG between 1 January 2010 and 31 June 2016. All patients were divided into two groups: Group 1 comprised 6396 CABG patients operated on with the off-pump technique. Group 2 included 1210 patients who received CABG with the on-pump technique. Preoperative data were collected on the studied variables. Postoperative data included the development of intestinal ischemia and in-hospital mortality.

**Results:**

Of 7606 consecutive CABG patients, a total of 31 (0.4%) developed intestinal ischemia. The incidence of postoperative mesenteric ischemia was 0.28% in Group 1 and 1.07% in Group 2 (P = 0.000). The survival rates after AMI were 61.1% in Group 1 (off-pump) and 7.7% in Group 2 (on-pump) (P = 0.003). Time from the first occurrence of nonspecific GI complaints to laparotomy was similar in the off-pump and on-pump groups and had no effect on mortality.**Conclusions:** With regard to the incidence of mesenteric ischemia and survival after laparotomy, off-pump CABG patients revealed significant improvement compared with those operated on with the on-pump technique.

## 1. Introduction 

Acute mesenteric ischemia (AMI) is a rare but highly fatal complication of coronary artery bypass grafting (CABG). It has been attributed to perioperative low cardiac output and visceral hypoperfusion resulting in mucosal ischemia and necrosis. It has been speculated that, despite the normal indices of global perfusion, cardiopulmonary bypass (CPB) can be hazardous to mucosal ischemia by reducing the blood flow and stimulating the systemic inflammatory response (SIR), secondary to mesenteric sequestration of neutrophils (1–3). Off-pump CABG, in contrast, has been reported to reduce SIR, allowing an environment that is physiologically more favorable for the organ systems (4). Moreover, the off-pump technique reduces the need for systemic vasoconstrictors or inotropic requirements during cardiac surgery (5). However, the role of on-pump or off-pump surgery on the occurrence of AMI following CABG has not been made clear yet. 

In the present study, we aimed to compare the incidence and mortality rate of mesenteric ischemia in a series of consecutive patients undergoing CABG through on-pump and off-pump techniques. 

## 2. Materials and methods

This single-center study was approved by the Güven Hospital Ethics Committee and conducted in accordance with the principles of the Declaration of Helsinki. Written informed consent was obtained from each patient. We selected patients who underwent CABG surgery between 1 January 2010 and 31 June 2016 from the cardiovascular-thoracic database of Güven Hospital, Ankara. Patients with concomitant procedures, such as valve repair and valve replacement, were excluded from the study. All patients were divided into two groups: Group 1 consisted of 6396 CABG patients operated on with the off-pump technique. Group 2 included 1210 patients who received CABG with the on-pump technique. All operations were performed by a single surgical team. 

Preoperative data were collected on the following variables: age, sex, family history, angina class, urgency of operation, smoking, body mass index, diabetes, hypertension, prior cardiac surgery, history of myocardial infarction, recent myocardial infarction, hypercholesterolemia, peripheral vascular disease, chronic obstructive pulmonary disease, cerebrovascular disease, previous gastrointestinal surgery, renal dysfunction, the extent of coronary disease, and left ventricular ejection fraction. Data on the use of CPB, duration of CPB, and aortic cross-clamp were also collected*. *

Postoperative data included the development of intestinal ischemia, in-hospital mortality, reexploration for bleeding, atrial arrhythmia, prolonged mechanic ventilation, hemofiltration, atrial fibrillation, renal failure, highest creatine kinase-MB (CK-MB) release on postoperative day 1, cardiogenic shock, and use of inotropes and intraaortic balloon pumps.

Prolonged ventilator time was defined as the use of a ventilator for more than 24 h after cardiac surgery. The need for inotropic support was registered if the patient required one or more inotropic drugs, e.g., norepinephrine, dobutamine, and dopamine, for more than 24 h. Postoperative renal failure was defined as a serum creatinine level higher than 2 mg/dL. Intestinal ischemia was defined as ischemia diagnosed at endoscopy and abdominal surgery. Cardiac infarction was defined as the elevation of cardiac enzymes or electrocardiographic changes after the cardiac operation.

Urgent exploratory laparotomy was performed if any peritoneal signs occurred in any patient at any time during evaluation. Standard surgical therapy for AMI involved intestinal resection with anastomosis or enterostomy if necrotic bowel segments were found, and a second-look procedure was planned for doubtful cases 24 h after resection of irreparably damaged bowel. 

Cardiopulmonary bypass was maintained with nonpulsatile blood flow between 2.0 and 2.2 L (min m2) body surface area with a mean perfusion pressure between 70 and 80 mmHg and systemic cooling to 28 to 32 °C. The ascending aorta, or occasionally the femoral artery, was used for arterial cannulation and right atrial or bicaval cannulation for the venous return. Cold, intermittent, anterograde, and retrograde blood or crystalloid cardioplegia with or without topical cooling were the methods of myocardial protection.

Statistical analysis was performed using the SPSS 23.0 (IBM Corp., Armonk, NY, USA). Descriptive data were expressed as frequency, percentage, mean, and standard deviation. The chi-square (χ2) test was used to compare qualitative data. The compliance of data with normal distribution was assessed using the Kolmogorov–Smirnov test and the Shapiro–Wilk test. The independent samples t-test was used to analyze normally distributed quantitative data. P < 0.05 was considered statistically significant. 

## 3. Results

Of a total of 7606 consecutive CABG patients, 31 patients (0.4%) developed intestinal ischemia. All patients who underwent laparotomy were diagnosed with intestinal ischemia.

The demographic and clinical characteristics the and pre- and postoperative data of all patients receiving on-pump and off-pump techniques are shown in Table 1. The EuroSCORE value and steroid and inotropic agent use were found to be significantly higher in the patients with AMI operated on-pump (P < 0.05).

**Table 1:** Clinical characteristics of all patients with mesenteric ischemia.

		Off-pump(n=18)	On-pump(n=13)	P
Age		70.5 ± 9.8	67.5 ± 10.5	0.414*
EuroSCORE		3.7 ± 1.9	5.8 ± 2.9	0.020*
Sex	Female	2 (11.1%)	5 (38.5%)	0.099**
	Male	16 (88.9%)	8 (61.5%)	
Diabetes mellitus	Absent	13 (72.2%)	8 (61.5%)	0.701**	
	Present	5 (27.8%)	5 (38.5%)	
Hypertension	Absent	4 (22.2%)	4 (30.8%)	0.689**
	Present	14 (77.8%)	9 (69.2%)	
Current smoking	Absent	10 (55.6%)	9 (69.2%)	0.438**
	Present	8 (44.4%)	4 (30.8%)	
Family history	Absent	6 (33.3%)	7 (53.8%)	0.253**
	Present	12 (66.7%)	6 (46.2%)	
Hemofiltration	Absent	15 (83.3%)	11 (84.6%)	1.000**
	Present	3 (16.7%)	2 (15.4%)	
Chronic renal failure	Absent	15 (83.3%)	11 (84.6%)	1.000**	
Present	3 (16.7%)	2 (15.4%)		
Urgent operation	Absent	18 (100.0%)	12 (92.3%)	0.419**
	Present	0 (0.0%)	1 (7.7%)	
Cerebrovascular disease	Absent	14 (77.8%)	12 (92.3%)	0.368**
	Present	4 (22.2%)	1 (7.7%)	
Intraaortic balloon pump	Absent	17 (94.4%)	11 (84.6%)	0.558**
	Present	1 (5.6%)	2 (15.4%)	
COPD***	Absent	17 (94.4%)	13 (100.0%)	1.000**
	Present	1 (5.6%)	0 (0.0%)	
Peripheral vascular disease	Absent	15 (83.3%)	12 (92.3%)	0.621**
	Present	3 (16.7%)	1 (7.7%)	
Prolonged mechanic ventilation	Absent	17 (94.4%)	12 (92.3%)	1.000**
	Present	1 (5.6%)	1 (7.7%)	
Previous cardiac surgery	Absent	18 (100.0%)	12 (92.3%)	0.419**
	Present	0 (0.0%)	1 (7.7%)	
Prior myocardial infarction	Absent	13 (72.2%)	10 (76.9%)	1.000**
	Present	5 (27.8%)	3 (23.1%)	
Inotropic agents/vasopressors	Absent	14 (77.8%)	2 (15.4%)	0.000**
	Present	4 (22.2%)	11 (84.6%)	
Cardiogenic shock	Absent	17 (94.4%)	10 (76.9%)	0.284**
	Present	1 (5.6%)	3 (23.1%)	
Steroid	Absent	17 (94.4%)	5 (38.5%)	0.000**
	Present	1 (5.6%)	8 (61.5%)	
Arrhythmia	Absent	17 (94.4%)	11 (84.6%)	0.558**
	Present	1 (5.6%)	2 (15.4%)	
Chronic atrial fibrillation	Absent	9 (50.0%)	7 (53.8%)	0.832**
	Present	9 (50.0%)	6 (46.2%)	

*Independent samples t-test. **Chi-square test. ***Chronic obstructive pulmonary disease.

The incidence of postoperative mesenteric ischemia was 0.28% in Group 1 and 1.07% in Group 2 (P = 0.000) (Table 2). 

**Table 2:** Rate of mesenteric ischemia in on- or off-pump groups of CABG patients.

		Group 1(Off-pump)	Group 2 (On-pump)	Total	P*
Mesenteric ischemia
	Absent	6378	1197	7575	0.000
	Present	18	13	31	
	Total	6396	1210	7606	

*Chi-square test.

The types of surgery performed in 31 patients who developed mesenteric ischemia are listed in Table 3. One patient was considered as unresectable due to the presence of massive ischemia.

**Table 3:** Bowel surgery procedures.

Procedure	n
Right hemicolectomy	17
Right hemicolectomy + Subtotal small bowel resection	7
Subtotal small bowel resection	4
Subtotal colectomy	2
Unresectable	1

The overall in-hospital mortality rate for CABG in our institution during the study time period was 3.5% (264/7606). Of these cases, 19 (7.1%) were due to acute mesenteric ischemia and its complications. As shown in Table 4, survival rates after AMI were 61.1% in Group 1 (off-pump) and 7.7% in Group 2 (on-pump) (P = 0.003). 

**Table 4:** Survival rates in on- and off-pump surgery patients with acute mesenteric ischemia (n = 31).

	Off-pump	On-pump	P
Dead	7	12	0.003
Alive	11	1	
Total	18	13	

*Chi-square test.

The time from the first occurrence of nonspecific GI complaints to laparotomy was similar in the off-pump and on-pump groups (Table 5) and had no effect on mortality (Table 6). 

**Table 5:** Time (days) between symptoms and laparotomy in AMI patients.

	Off-pump(n = 18)	On-pump (n = 13)	P
Symptom to laparotomy time (days)	2.4 ± 0.9	2.3 ± 1.7	0.923

*Independent samples t-test.

**Table 6:** Time (days) between symptoms and laparotomy according to survival of AMI patients.

	Dead(n=19)	Alive(n=12)	P*
Symptom to laparotomy time (days)	2.3 ± 1.4	2.3 ± 0.9	0.970

*Independent samples t-test.

## 4. Discussion 

In the present study, based on a large prospectively collected database, mesenteric ischemia after CABG surgery was investigated. In our series, 31 of 7606 (0.4%) patients were found to suffer from AMI after CABG. Our results were correlated with the results from the literature (0.07%–0.5%) (6). We also found* *that the incidence of mesenteric ischemia varied according to the use of on- or off-pump technique during CABG. Patients in the on-pump group had a significantly higher incidence of AMI than those in the off-pump group (Table 2).

For the past two decades, the use of CPB during CABG has been challenged by the off-pump technique. There have been numerous reports comparing the results of on- and off-pump surgery in terms of morbidity and mortality (7–12); however, comparative studies focusing on mesenteric ischemia are still limited. 

Mesenteric ischemia is a rare but highly fatal complication after CABG (13–15). Risk factors for mesenteric ischemia include advanced age, intraoperative hypoperfusion, emergency operation, the need for high-dose vasopressors, longer CPB times, and intraaortic balloon pumps (13,14,16–18).

Despite the normal indices of global perfusion, CPB has been shown to cause a reduction in mucosal blood flow, lead to mesenteric sequestration of neutrophils, and stimulate the SIR. Vasoconstrictor factors released during CPB lead to redistribution of the blood flow away from the mucosa because of regional vasoconstriction, thereby contributing to mucosal ischemia (19,20). In our series, among 31 patients with AMI, the use of inotropic and vasoconstrictor agents was significantly higher in the on-pump than off-pump surgery cases (84.6% vs. 22.2%, P = 0.000).

Undergoing a laparotomy and major gastrointestinal surgery soon after CABG may be a highly morbid and mortal procedure. In the present study, a higher number of the patients were operated on for AMI in on-pump than off-pump surgeries (P = 0.000). In addition, a higher number of patients in the on-pump group died after gastrointestinal surgery compared to off-pump (P = 0.003). As the demographic and clinical variables, including extracardiac risk factors and the time from symptom to laparotomy, were similar in the two groups, one possible explanation for this finding may be the unfavorable status related to the pump and its consequences. 

There are several limitations to our study. First, being a retrospective database study, by its nature it is only capable of showing associations between variables and outcomes and is unable to demonstrate cause and effect. Second, the incidence of the complication is very low. Consequently, in this study, only 31 patients could be identified who could be matched. A larger sample size could have allowed more meaningful statistical comparisons and multivariate analysis so that risk models could be built. However, this study has the advantage of being a single center, single cardiac surgeon study, thus greatly reducing the effects of different surgical techniques and perioperative or postoperative protocol.

In conclusion, regarding the incidence of mesenteric ischemia and survival after laparotomy, our study results suggest that off-pump CABG patients achieve significant improvement compared to those undergoing on-pump surgery.
